# Live fast die young life history in females: evolutionary trade-off between early life mating and lifespan in female *Drosophila melanogaster*

**DOI:** 10.1038/srep15469

**Published:** 2015-10-20

**Authors:** Laura M. Travers, Francisco Garcia-Gonzalez, Leigh W. Simmons

**Affiliations:** 1 Centre for Evolutionary Biology, School of Animal Biology (M092), The University of Western Australia, Crawley, WA 6009, Australia; 2 Doñana Biological Station, Spanish Research Council CSIC, c/ Americo Vespucio, s/n, Isla de la Cartuja 41092, Sevilla, Spain

## Abstract

The trade-off between survival and reproduction is fundamental to life history theory. Sexual selection is expected to favour a ‘live fast die young’ life history pattern in males due to increased risk of extrinsic mortality associated with obtaining mates. Sexual conflict may also drive a genetic trade-off between reproduction and lifespan in females. We found significant additive genetic variance in longevity independent of lifetime mating frequency, and in early life mating frequency. There was significant negative genetic covariance between these traits indicating that females from families characterized by high levels of multiple mating early in life die sooner than females that engage in less intense early life mating. Thus, despite heritable variation in both traits, their independent evolution is constrained by an evolutionary trade-off. Our findings indicate that, in addition to the well-known male-driven direct costs of mating on female lifespan (mediated by male harassment and harmful effects of seminal fluids), females with a genetic propensity to mate multiply live shorter lives. We discuss the potential role of sexual conflict in driving the evolutionary trade-off between reproduction and lifespan in *Drosophila*. More generally, our data show that, like males, females can exhibit a live fast die young life history strategy.

Senescence is defined as decreasing reproductive performance and increasing probability of death with age^1^. Theory proposes that senescence evolves as a result of decreased selection on genes expressed late in life as few individuals reach old age due to extrinsic mortality factors^2^,^3^,^4^,^5^. The Disposable Soma theory suggests that trade-offs result from the partitioning of finite resources between growth, maintenance and reproduction^5^. These trade-offs are important because they enable the accumulation of alleles with deleterious effects that are only expressed late in life (mutation accumulation^2^) and/or cause antagonistic pleiotropic effects on alleles, where genes that have positive effects on fitness early in life have deleterious effects on later survival (antagonistic pleiotropy^3^). Such alleles have the potential to generate genetic trade-offs among life history traits and constrain their independent evolution^3^,^6^.

The trade-off between survival and reproduction has long been recognised as a prominent feature of life history trajectories, and it is well established that reproduction reduces lifespan in many species^7^. Our understanding of the evolution of ageing, and how reproduction affects it has advanced greatly from research on *Drosophila melanogaster*. Numerous artificial selection experiments in this species provide evidence supporting a trade-off between early fecundity and late fecundity or survival. For example, artificial selection by Rose^8^ found higher rates of egg laying in late life than early life in females selected for extended longevity compared to control lines, and Partridge *et al.*^9^ found a decrease in fertility early in life in lines selected for longer life. Other studies have also found correlated responses to selection for late fecundity with increased lifespan in *D. melanogaster*^10^,^11^,^12^. The general finding of lifespan and fertility increasing in lines propagated from old adults, and pre-adult survival or fecundity of young adults showing a correlated decline with lifespan show that trade-offs are important in the evolution of ageing in this model system.

Sexual selection influences the costs of reproduction and the benefits of early versus late reproduction for each sex. Bateman’s principle predicts that males can increase their fitness by mating with many females, while females, due to a limitation on the number of eggs they can produce and their typically larger parental investment, are expected to maximise their reproductive success with only one or a few matings^13^,^14^. The dichotomy in reproductive investment between the sexes means that males typically allocate more resources to competition for matings than females^14^. Males are more likely to suffer increased mortality due to costly secondary sexual traits (costly in terms of energy expenditure and increased predation) and direct injury in combat competition to gain access to mates. Consequently, males are expected to pursue high risk ‘live fast, die young’ life history strategies that have potential to yield high fitness returns over short time periods^15^,^16^. Hence, it is predicted that selection will favour male reproductive strategies that sacrifice longevity for mating opportunities^15^,^17^. Indeed, empirical findings suggest that male mortality is higher than female mortality across a range of taxa^18^. In contrast, because females are limited by the time and energy requirements for offspring production, selection acting on females is expected to promote low risk, low wear and tear strategies with moderate rates of return over extended time periods^16^. In support of this hypothesis, male crickets selected for decreased lifespan increase early reproductive performance (calling), whereas female longevity was reduced without influencing their reproductive success^19^.

According to evolutionary theories of ageing, increased rates of extrinsic mortality should lead to accelerated rates of intrinsic mortality^4^, which reduces selection on late life performance^20^. This has led to the recognition that antagonistic coevolution between the sexes may promote an evolutionary trade-off between reproduction and lifespan. While evidence is mounting for diverse benefits of female multiple mating across species^21^,^22^,^23^, it is also well established that mating incurs a cost to females, and the archetypical example is *D. melanogaster*^24^,^25^,^26^,^27^,^28^,^29^,^30^,^31^. The increase in female mortality caused by sexually antagonistic adaptations in males should result in the accumulation of alleles with deleterious late life effects. Promislow^32^ suggests higher rates of sexual conflict lead to the evolution of higher rates of senescence. Maklakov *et al.*^33^ found the removal of sexual conflict via enforced monogamy in populations of the seed beetle *Callosobruchus maculatus* led to increased lifespan in virgin females from monogamous populations compared to polygamous populations. However, sexual conflict could also result in selection for increased somatic maintenance to repair the damage induced by toxic ejaculates and hence increase lifespan^33^,^34^. For example, Reznick, *et al.*^35^ found that higher extrinsic mortality mediated by predation in *Poecilia reticulata* guppies was associated with decelerated rates of intrinsic mortality. Promislow *et al.*^36^ also found that increased opportunity for sexual selection was genetically correlated with adult survivorship in *D. melanogaster*. Therefore, antagonistic coevolution could theoretically promote either accelerated or decelerated ageing.

An important prediction of the mutation accumulation and antagonistic pleiotropy theories of life history evolution is that the trade-off between reproduction and lifespan leads to negative genetic correlations. Implicit in this prediction is the assumption of the presence of additive genetic variation in which genes that alter reproduction early in life simultaneously alter survival in late life^37^. In this study we aimed to investigate the genetic trade-off between female longevity and early life mating frequency. However, quantification of genetic variation in lifespan is not straightforward because of the phenotypic cost of mating due to sexual conflict. Using a quantitative genetic design, we calculated quantitative genetic estimates for female lifespan while controlling for lifetime mating frequency, and genetic estimates for early life mating frequency while controlling for longevity in a natural population of *D. melanogaster*. We examined the relationship between early life mating frequency and lifetime mating rate to confirm that our measure of early life mating frequency was indicative of lifetime mating frequency. We then investigated the potential for an evolutionary trade-off between lifespan and early life mating by measuring the genetic covariance between the two traits.

## Results

**Figure 1:**
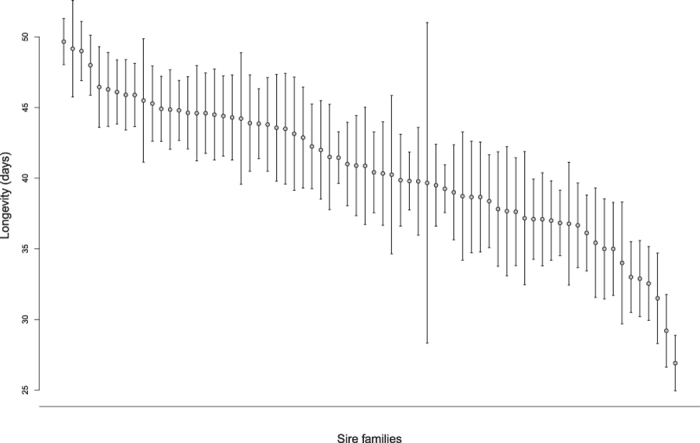
Raw sire family means of longevity.

We found substantial phenotypic variation in longevity (mean ± SD = 40.460 days ± 10.361; range = 13–62). Raw sire family means of longevity are displayed in Fig. 1. There was a significant effect of total number of lifetime matings on longevity (Wald χ^*2*^ = 74.279 df = 1, p < 0.001), with longer lived females having a higher total number of matings in their life. There was a significant effect of start date (Wald χ^*2*^ = 10.439 df = 1, p = 0.001) but no significant effect of body size (Wald χ^*2*^ = 0.147, df = 1, p = 0.702) on longevity. Our genetic estimates for longevity after controlling for the number of lifetime matings showed considerable levels of additive genetic variation and narrow sense heritability (Table 1) and revealed significant variance among sires (χ^*2*^ = 17.385, df = 1, p < 0.001).

Early life mating frequency also showed considerable phenotypic variation (mean ± SD = 2.085 ± 0.88). Neither longevity (Wald χ^*2*^ = 0.810, df = 1, p = 0.368), body size (Wald χ^*2*^ = 2.070, df = 1, p = 0.150) or start date (Wald χ^*2*^ = 3.348, df = 1, p = 0.067) had a significant effect on the measure. Early life mating frequency displayed high narrow sense heritability and additive genetic variance (Table 1), and significant sire variance (χ^*2*^ = 23.77, df = 1, p < 0.001).

**Figure 2:**
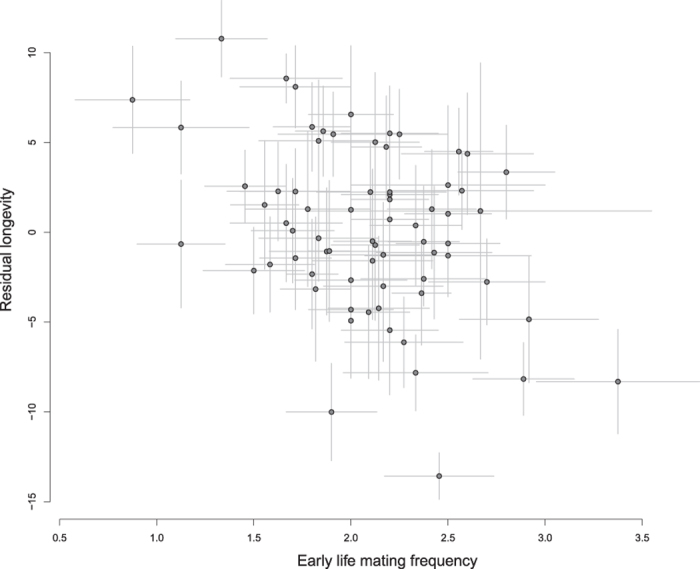
Sire family mean relationship between residual longevity and early life mating frequency. Residual longevity was obtained from a linear regression of longevity on lifetime mating frequency and start date. Points and error bars correspond to sire family means and standard errors, respectively.

We found a significant negative genetic covariance between early life mating frequency and longevity (r_g_ = −0.651, SE = 0.138). Figure 2 shows the relationship between sire family means of residual longevity and early life mating frequency. A significant positive correlation between early life and later life mating frequency was also found (r_g_ = 0.692, SE = 0.171).

## Discussion

Our analyses of lifespan and early life mating frequency showed strong negative genetic covariance, indicative of an evolutionary trade-off. Numerous artificial selection experiments have selected for altered lifespan in this species and found correlated responses indicative of a trade-off between reproductive effort (e.g. fecundity, egg viability, competitive larval viability) and lifespan^8^,^10^,^12^ (for review see^38^). Our study has quantified the additive genetic basis of traits underlying the trade-off between lifespan and early life mating and found high levels of genetic variance in both traits. The genetic trade-off indicates that alleles that have a positive effect on early life mating also have a negative effect on longevity, as predicted by evolutionary theories of senescence. Additive genetic variation in these traits may in part be maintained by the opposing effects of their alleles on female fitness. Such alleles are likely to remain for longer periods at intermediate frequencies within a population, compared to alleles that have a positive effect on both traits whereby directional selection is expected to erode genetic variation^37^.

The negative genetic correlation between early life mating frequency and longevity is indicative of variation in female life history strategies. Male reproductive strategies are typically associated with elevated mortality risks and weaker selection for long lifespan compared to females, due to the high cost of bearing secondary sexual traits and higher extrinsic mortality^16^. For example, Robinson, *et al.*^39^ found a trade-off between annual breeding success and longevity in males, with larger horn size associated with reduced longevity in a population of Soay sheep. Lemaître, *et al.*^40^ also found increased rates of ageing in male red deer that controlled larger harems and rutted for longer periods which suggests males that invest more energy in reproduction early in life age at a faster rate. In females, Charmantier, *et al.*^41^ found a genetic trade-off between age at first reproduction and age at last reproduction in a population of free ranging mute swans (*Cygnus olor*), suggesting that increased early life performance trades off with earlier reproductive senescence. However, Bérubé *et al.*^42^ found no trade-off between female longevity and early life reproduction in two wild living populations of bighorn sheep (*Ovis canadensis*). We found that females from families characterized by high levels of multiple mating early in life died sooner than females from families that engaged in less intense early life mating. Our results thereby support the live fast die young trade-off in females. The positive correlation between early and late life mating suggests that our early life measure of mating frequency reflects overall lifetime mating strategy. Hence, variation in reproductive strategies within this population may constrain the evolution of lifespan.

The relationship between extrinsic mortality and lifespan is common to the main evolutionary explanations of senescence. Accordingly, it is now recognised that damage caused by sexual conflict may play an important role in the evolution of sex specific rates of mortality^16^. Importantly, our results show a genetic trade-off between early life mating and lifespan that is independent of the phenotypic effect of male seminal fluids on female lifespan. Therefore, in addition to the harmful effects of male seminal fluids on lifespan, our findings suggest that females with a genetic propensity to mate multiply live shorter lives. Sexual conflict in this species could contribute to the accumulation of alleles with deleterious effects in old age or accumulation of alleles that enhance early life fitness at the cost of late life fitness. It is possible that elevated rates of mortality in females due to toxic male seminal fluid proteins could drive a live fast die young strategy in females, due to weakened selection on somatic maintenance. The optimal strategy favoured by natural selection might be high rates of mating in early life because females would be unlikely to survive long enough in natural populations of flies to experience fitness costs associated with deleterious alleles expressed in late life. The intensity of female mating frequency may in part reflect a balance between the benefits to females of mating multiply and the cost of reduced lifespan. Our findings suggest that antagonistic coevolution not only imposes a phenotypic cost to mating but that it could potentially drive the evolution of lifespan of females in this species.

## Methods

We used a full sib half sib breeding design to quantify genetic variation in female adult lifespan and lifetime mating frequency for 775 daughters distributed among 72 sire families and 198 dam families of *D. melanogaster*. Focal flies came from a laboratory population of sixth generation descendants of wild type *D. melanogaster* collected near Innisfail in Northern Queensland, Australia. To produce parents of focal females, grape agar plates were placed in the population cage for 4 h. The following day, we collected first instar larvae and transferred them to vials at a standard density of 50 larvae per vial. Vials contained 10 ml of sugar-maize medium. Offspring were collected 9–11 days later under CO_2_ anaesthesia within 8 h of eclosion and transferred to single sex vials. Males were kept at a density of 10 per vial and females at 5 per vial.

Parental generation matings were carried out when flies were 3–4 days old. Each male was mated to three virgin females to generate families of paternal half siblings and maternal full siblings. After mating, females were transferred to individual vials and moved to new vials every 48 h for four days. During peak eclosion, eight virgin female offspring (daughters) from each full sibling dam family were randomly collected; four were included in the lifetime mating frequency assay and four were frozen and later used to estimate full sibling dam family average female body size. Mating opportunities for daughters began at 3–5 days of age. Females were kept in individual vials and transferred to fresh food vials every week. Each female was given a mating opportunity with a sexually naïve male every Monday, Wednesday and Friday over her entire lifespan. Successful copulations were recorded and all pairs separated after 90 mins.

It is well known in this species that male seminal fluid proteins transferred during mating reduce female survival through their toxic effects^25^,^43^,^44^. It is also well established that male seminal fluid proteins mediate female remating^27^,^43^. Therefore, male effects are likely to introduce additional environmental variation to our genetic estimates of lifespan and female mating frequency. To address this problem we estimated the additive genetic variance of female mating frequency and lifespan by using standardized males as mating partners (see^45^ for rationale). Hence, to reduce male induced variation in female lifespan and female remating, for each mating opportunity, we standardised the male identity by randomly selecting naïve males from one of 10 isogenic lines. Each isogenic line had been generated through full sibling matings started with one founder pair taken from a replica of a LH_M_ population^46^ (see below). We used 2–3 day old males for the mating trials. The standardization results in more precise estimates of polyandry by minimizing sampling variance induced by randomly selecting mates that differ in their effects on female survival and remating propensity.

Male isogenic lines were obtained through full sibling matings for 16 generations, followed by several generations of within line matings (approx. 15 individuals from each vial for each new generation). Full sibling matings were then reinstated for another 21 generations. Isogenic lines were then mass bred into population cages to allow collection of an adequate numbers of flies needed for mating trials. We standardized the identity of the male partner by randomly selecting males from the same isogenic line in each mating opportunity. Larvae were collected from population cages on grape agar plates and sexually naïve males collected at peak eclosion.

### Statistical Analyses

To investigate the genetic basis of longevity and early life mating frequency, a subset (n = 613) was generated from the full dataset to include only females (daughters from the full sib half sib breeding design) that lived long enough to have at least six mating opportunities and that mated at least once. All females had a different number of mating opportunities throughout their life, depending on how long they lived. The rationale for subsetting the data to include a fixed number of mating opportunities (first six opportunities) was to control for the possible confounding effect of females with more mating opportunities having a lower lifetime remating proportion. Such a confounding effect may arise due to age related decline in mating rate, and increased probability of males failing to attempt courtship with females having had more mating opportunities. Furthermore, it is unlikely that females with a large number of mating opportunities will maintain high remating rates throughout all opportunities compared to females with only few mating opportunities. The subset also eliminated females that escaped or were accidentally killed. Thus, only females that had lived a ‘natural’ lifespan were included. The lme4 package^47^ implemented in R 3.03.3^48^ was used to fit standard nested mixed models for a paternal half-sibling design. A Linear Mixed Model (LMM) on untransformed longevity was fitted, after residuals were tested for normal distribution, using the lmer function with sire and dam nested within sire as random effects. A LMM was fitted on log (x + 1) transformed number of matings accepted in the first six opportunities, hereafter referred to as early life mating frequency, using the lmer function with sire and dam nested within sire as random effects. All females were given the first opportunity to mate when they were 3–5 days old but for logistical reasons there was variation among sire and dam families in the date we started the assays. To control for potential temporal variation in longevity and early life mating frequency, we included assay start date as a fixed effect in both analyses. We also included total lifetime mating frequency as a fixed effect in our genetic analysis of longevity to control for the effect of mating frequency on lifespan. Longevity was included as a covariate in the genetic analysis of early life mating frequency. We also investigated the effect of body size on both traits. Significance of fixed effects was tested using Wald chi-square tests implemented in the Anova function of the car package^49^. Significance of the sire and dam variance components were determined using likelihood-ratio tests.

Genetic parameters for both traits were calculated using restricted maximum likelihood (REML) from LMMs using the lmer function. We performed the analyses on untransformed data because many genetic parameters (e.g. CV_A_ and I_A_) cannot be used for comparative purposes if variance components are extracted when data are transformed^50^. Observational variance components were estimated from minimal models including only significant fixed effects. Narrow sense heritabilities (*h*^*2*^) were estimated from the ratio of additive genetic variance (V_A_: four times the sire variance component) to total phenotypic variance. Mean-standardized measures of evolvability were calculated, namely the coefficient of additive genetic variation (CV_A_), and I_A_^50^,^51^,^52^. CV_p_ and CV_r_ were also calculated as described in^50^. Standard errors for all quantitative genetic parameters were calculated by jackknifing across sire families^53^.

We used bivariate Animal Models in ASREML 3^54^ to examine the genetic correlation between early life (number of matings accepted in opportunities 1–6) and later life mating frequency (number of matings accepted in opportunities 7- death). We also calculated the genetic correlation between longevity and early life mating frequency with bivariate animal models.

## Additional Information

**How to cite this article**: Travers, L. M. *et al.* Live fast die young life history in females: evolutionary trade-off between early life mating and lifespan in female *Drosophila melanogaster*. *Sci. Rep.* **5**, 15469; doi: 10.1038/srep15469 (2015).

## Figures and Tables

**Table 1:** Quantitative genetic parameters for longevity (after controlling for lifetime mating frequency) and early life mating frequency.

	N	Mean (SE)	n sires	n dams	V_Sire_ (SE)	V_Dam_ (SE)	V_A_ (SE)	V_P_ (SE)	V_R_ (SE)	*h*^2^ (SE)	CV_A_(SE)	CV_P_(SE)	CV_R_ (SE)	I_A_ (SE)	P_Sire_	P_Dam_
Longevity	613	40.460 (0.420)	70	197	12.602 (4.540)	0	50.410 (18.161)	96.393 (4.793)	83.791 (4.867)	0.523 (0.180)	0.175 (0.034)	0.243 (0.008)	0.168 (0.029)	0.031 (0.011)	<0.001*	1
Early life mating frequency	613	2.085 (0.035)	70	197	0.122 (0.039)	0.002 (0.016)	0.487 (0.156)	0.644 (0.048)	0.644 (0.048)	0.634 (0.176)	0.335 (0.059)	0.420 (0.018)	0.254 (0.049)	0.112 (0.037)	<0.001*	0.295

Number of offspring (N), trait means (longevity in days), number of sire (half-sib) and dam (full-sib) families (n), variance components for sires (V_Sire_) and dams (V_Dam_), additive genetic variation (V_A_), total phenotypic variation (V_p_), residual variation (V_R_), narrow sense heritabilities (*h*^*2*^), mean-standardized additive genetic variances (Evolvabilities: CV_A_ and I_A_), coefficient of phenotypic variation CV_p_, coefficient of residual variation CV_R_, and significance values for Sire and Dam effects (P_Sire_ and P_Dam_). Standard errors (SE) are provided within brackets.

## References

[b1] Rose M. R. Evolutionary biology of aging. Vol. 164 (Oxford University Press New York, 1991).

[b2] Medawar P. B. Aging: An Unsolved Problem of Biology. (HK Lewis, 1952).

[b3] Williams G. C. Pleiotropy, natural selection, and the evolution of senescence. Evolution 11, 398–411 (1957).

[b4] Partridge L. & Barton N. Evolution of aging: Testing the theory using Drosophila. Genetica 91, 89–98 (1993). 8125281 10.1007/BF01435990

[b5] Kirkwood T. B. Evolution of ageing. Nature 270, 301–304 (1977). 593350 10.1038/270301a0

[b6] Kirkwood T. B. L. & Rose M. R. Evolution of senescence: late survival sacrificed for reproduction. Philosophical Transactions of the Royal Society of London. Series B, Biological sciences. 332, 15–24 (1991). 10.1098/rstb.1991.0028 1677205

[b7] Stearns S. C. The evolution of life histories. (Oxford University Press Oxford, 1992).

[b8] Rose M. R. Laboratory evolution of postponed senescence in Drosophila melanogaster. Evolution 38, 1004–1010 (1984). 10.1111/j.1558-5646.1984.tb00370.x 28555803

[b9] Partridge L., Prowse N. & Pignatelli P. Another set of responses and correlated responses to selection on age at reproduction in *Drosophila melanogaster*. Proceedings of the Royal Society of London B: Biological Sciences 266, 255–261 (1999). 10.1098/rspb.1999.0630 PMC1689678 10081162

[b10] Rose M. R. & Charlesworth B. Genetics of life history in *Drosophila melanogaster* II. Exploratory selection experiments. Genetics 97, 187–196 (1981). 6790341 10.1093/genetics/97.1.187 PMC1214383

[b11] Luckinbill L. S., Arking R., Clare M. J., Cirocco W. C. & Buck S. A. Selection for delayed senescence in *Drosophila melanogaster*. Evolution 38, 996–1003 (1984). 10.1111/j.1558-5646.1984.tb00369.x 28555795

[b12] Partridge L. & Fowler K. Direct and correlated responses to selection on age at reproduction in *Drosophila melanogaster*. Evolution 46, 76–91 (1992). 10.1111/j.1558-5646.1992.tb01986.x 28564973

[b13] Bateman A. J. Intra-sexual selection in *Drosophila*. Heredity 2, 349 (1948). 18103134 10.1038/hdy.1948.21

[b14] Trivers R. In Parental investment and sexual selection. Sexual Selection & the Descent of Man 136–179 (Aldine Publishing Company, 1972).

[b15] Vinogradov A. E. Male reproductive strategy and decreased longevity. Acta biotheoretica 46, 157–160 (1998). 9691260 10.1023/a:1001181921303

[b16] Bonduriansky R., Maklakov A., Zajitschek F. & Brooks R. Sexual selection, sexual conflict and the evolution of ageing and life span. Functional Ecology 22, 443–453, 10.1111/j.1365-2435.2008.01417.x (2008).

[b17] Carranza J. & Pérez-Barbería F. J. Sexual selection and senescence: male size‐dimorphic ungulates evolved relatively smaller molars than females. The American Naturalist 170, 370–380 (2007). 10.1086/519852 17879188

[b18] Finch C. Senescence, longevity, and the genome. (University of Chicago Press, Chicago, 1990).

[b19] Hunt J., Jennions M. D., Spyrou N. & Brooks R. Artificial selection on male longevity influences age‐dependent reproductive effort in the Black field cricket *Teleogryllus commodus*. The American Naturalist 168, E72–E86 (2006). 10.1086/506918 16947102

[b20] Stearns S., Ackermann M., Doebeli M. & Kaiser M. Experimental evolution of aging, growth, and reproduction in fruitflies. Proceedings of the National Academy of Sciences 97, 3309–3313 (2000). 10.1073/pnas.060289597 PMC16235 10716732

[b21] Arnqvist G. & Nilsson T. The evolution of polyandry: multiple mating and female fitness in insects. Animal Behaviour 60, 145–164 (2000). 10973716 10.1006/anbe.2000.1446

[b22] Newcomer S. D., Zeh J. A. & Zeh D. W. Genetic benefits enhance the reproductive success of polyandrous females. Proceedings of the National Academy of Sciences 96, 10236–10241 (1999). 10.1073/pnas.96.18.10236 PMC17872 10468592

[b23] Slatyer R. A., Mautz B. S., Backwell P. R. Y. & Jennions M. D. Estimating genetic benefits of polyandry from experimental studies: a meta-analysis. Biological Reviews 87, 1–33, 10.1111/j.1469-185X.2011.00182.x (2012). 21545390

[b24] Fowler K. & Partridge L. A cost of mating in female fruitflies. Nature 338, 760–761 (1989).

[b25] Chapman T., Liddle L. F., Kalb J. M., Wolfner M. F. & Partridge L. Cost of mating in *Drosophila melanogaster* females is mediated by male accessory gland products. Nature 373, 241–244 (1995). 7816137 10.1038/373241a0

[b26] Chapman T. Seminal fluid‐mediated fitness traits in *Drosophila*. Heredity 87, 511–521 (2001). 11869341 10.1046/j.1365-2540.2001.00961.x

[b27] Wolfner M. The gifts that keep on giving: physiological functions and evolutionary dynamics of male seminal proteins in *Drosophila*. Heredity 88, 85–93 (2002). 11932766 10.1038/sj.hdy.6800017

[b28] Rice W. R. Sexually antagonistic male adaptation triggered by experimental arrest of female evolution. Nature 381, 232–234 (1996). 8622764 10.1038/381232a0

[b29] Holland B. & Rice W. R. Experimental removal of sexual selection reverses intersexual antagonistic coevolution and removes a reproductive load. Proceedings of the National Academy of Sciences of the United States of America 96, 5083–5088, 10.1073/pnas.96.9.5083 (1999). 10220422 PMC21820

[b30] Pitnick S., Brown W. D. & Miller G. T. Evolution of female remating behaviour following experimental removal of sexual selection. *Proceedings of the Royal Society of London*. Series B: Biological Sciences 268, 557–563 (2001). 10.1098/rspb.2000.1400 PMC1088640 11297171

[b31] Pitnick S. & Garcia–Gonzalez F. Harm to females increases with male body size in *Drosophila melanogaster*. *Proceedings of the Royal Society of London*. Series B: Biological Sciences 269, 1821–1828 (2002). 10.1098/rspb.2002.2090 PMC1691094 12350270

[b32] Promislow D. Mate choice, sexual conflict, and evolution of senescence. Behavior genetics 33, 191–201 (2003). 14574152 10.1023/a:1022562103669

[b33] Maklakov A. A., Fricke C. & Arnqvist G. Sexual selection affects lifespan and aging in the seed beetle. Aging Cell 6, 739–744, 10.1111/j.1474-9726.2007.00333.x (2007). 17725688

[b34] Williams P. D. & Day T. Antagonistic pleiotropy, mortality source interactions, and the evolutionary theory of senescence. Evolution 57, 1478–1488, 10.1111/j.0014-3820.2003.tb00356.x (2003). 12940353

[b35] Reznick D. N., Bryant M. J., Roff D., Ghalambor C. K. & Ghalambor D. E. Effect of extrinsic mortality on the evolution of senescence in guppies. Nature 431, 1095–1099 (2004). 15510147 10.1038/nature02936

[b36] Promislow D. E., Smith E. A. & Pearse L. Adult fitness consequences of sexual selection in *Drosophila melanogaster*. Proceedings of the National Academy of Sciences 95, 10687–10692 (1998). 10.1073/pnas.95.18.10687 PMC27956 9724765

[b37] Reznick D. Costs of reproduction: an evaluation of the empirical evidence. Oikos 44, 257–267 (1985).

[b38] Harshman L. G. Life span extension of *Drosophila melanogaster*: genetic and population studies. Population and Development Review 29, 99–126 (2003).

[b39] Robinson M. R., Pilkington J. G., Clutton-Brock T. H., Pemberton J. M. & Kruuk L. E. Live fast, die young: trade‐offs between fitness components and sexually antagonistic selection on weaponry in Soay sheep. Evolution 60, 2168–2181 (2006). 17133873

[b40] Lemaître J.-F., Gaillard J.-M., Pemberton J. M., Clutton-Brock T. H. & Nussey D. H. Early life expenditure in sexual competition is associated with increased reproductive senescence in male red deer. Proceedings of the Royal Society B: Biological Sciences 281, 10.1098/rspb.2014.0792 (2014). PMC4150313 25122226

[b41] Charmantier A., Perrins C., McCleery R. H. & Sheldon B. C. Quantitative genetics of age at reproduction in wild swans: support for antagonistic pleiotropy models of senescence. Proceedings of the National Academy of Sciences 103, 6587–6592 (2006). 10.1073/pnas.0511123103 PMC1458927 16618935

[b42] Bérubé C. H., Festa-Bianchet M. & Jorgenson J. T. Individual differences, longevity, and reproductive senescence in Bighorn ewes. Ecology 80, 2555–2565, (1999).

[b43] Wolfner M. F. Tokens of love: Functions and regulation of *Drosophila* male accessory gland products. Insect Biochemistry and Molecular Biology 27, 179–192, 10.1016/s0965-1748(96)00084-7 (1997). 9090115

[b44] Lung O. *et al.* The *Drosophila melanogaster* seminal fluid protein Acp62F is a protease inhibitor that is toxic upon ectopic expression. Genetics 160, 211–224 (2002). 11805057 10.1093/genetics/160.1.211 PMC1461949

[b45] Garcia-Gonzalez F. & Evans J. P. Fertilization success and the estimation of genetic variance in sperm competitiveness. Evolution 65, 746–756, 10.1111/j.1558-5646.2010.01127.x (2011). 20880262

[b46] Byrne P. G. & Rice W. Remating in *Drosophila melanogaster*: an examination of the trading-up and intrinsic male-quality hypotheses. Journal of Evolutionary Biology 18, 1324–1331 (2005). 16135127 10.1111/j.1420-9101.2005.00918.x

[b47] Bates D., Maechler M. & Bolker B. lme4: Linear mixed-effects models using Eigen and S4. v. R package version 1.1-6. http://CRAN.R-project.org/package=lme4 (2014).

[b48] R Core Team: A language and environment for statistical computing. R Foundation for Statistical Computing, Vienna, Austria. URL http://www.R-project.org/. (2014).

[b49] Fox J. & Weisberg S. An R Companion to Applied Regression. Second Edition, (Thousand Oaks CA, 2011).

[b50] Garcia-Gonzalez F., Simmons L. W., Tomkins J. L., Kotiaho J. S. & Evans J. P. Comparing evolvabilities: common errors surrounding the calculation and use of coefficients of additive genetic variation. Evolution 66, 2341–2349 (2012). 22834736 10.1111/j.1558-5646.2011.01565.x

[b51] Houle D. Comparing evolvability and variability of quantitative traits. Genetics 130, 195–204 (1992). 1732160 10.1093/genetics/130.1.195 PMC1204793

[b52] Hansen T. F., Pélabon C. & Houle D. Heritability is not evolvability. Evolutionary Biology 38, 258–277 (2011).

[b53] Roff D. A. An introduction to computer-intensive methods of data analysis in biology. (Cambridge University Press, 2006).

[b54] ASReml user guide release 3.0 (VSN International Ltd, Hemel Hempstead, UK, 2009).

